# Alzheimer’s disease diagnosis using rhythmic power changes and phase differences: a low-density EEG study

**DOI:** 10.3389/fnagi.2024.1485132

**Published:** 2025-01-17

**Authors:** Juan Wang, Jiamei Zhao, Xiaoling Chen, Bowen Yin, Xiaoli Li, Ping Xie

**Affiliations:** ^1^Institute of Electrical Engineering, Yanshan University, Qinhuangdao, China; ^2^Key Laboratory of Intelligent Rehabilitation and Neuromodulation of Hebei Province, Qinhuangdao, China; ^3^Department of Neurology, The First Hospital of Qinhuangdao, Hebei Medical University, Qinhuangdao, China; ^4^State Key Laboratory of Cognitive Neuroscience and Learning & IDG/McGovern Institute for Brain Research, Beijing Normal University, Beijing, China; ^5^Center for Collaboration and Innovation in Brain and Learning Sciences, Beijing Normal University, Beijing, China

**Keywords:** Alzheimer’s disease, electroencephalography, resting state, low-density, Support vector machine

## Abstract

**Objectives:**

The future emergence of disease-modifying treatments for dementia highlights the urgent need to identify reliable and easily accessible tools for diagnosing Alzheimer’s disease (AD). Electroencephalography (EEG) is a non-invasive and cost-effective technique commonly used in the study of neurodegenerative disorders. However, the specific alterations in EEG biomarkers associated with AD remain unclear when using a limited number of electrodes.

**Methods:**

We studied pathological characteristics of AD using low-density EEG data collected from 26 AD and 29 healthy controls (HC) during both eye closed (EC) and eye opened (EO) resting conditions. The analysis including power spectrum, phase lock value (PLV), and weighted lag phase index (wPLI) and power-to-power frequency coupling (theta/beta) analysis were applied to extract features in the delta, theta, alpha, and beta bands.

**Results:**

During the EC condition, the AD group exhibited decreased alpha power compared to HC. Additionally, both analysis of PLV and wPLI in the theta band indicated that the alterations in the AD brain network predominantly involved in the frontal region with the opposite changes. Moreover, the AD group had increased frequency coupling in the frontal and central regions. Surprisingly, no group difference was found in the EO condition. Notably, decreased theta band functional connectivity within the fronto-central lobe and increased frequency coupling in frontal region were found in AD group from EC to EO. More importantly, the combination of EC and EO quantitative EEG features improved the inter-group classification accuracy when using support vector machine (SVM) in older adults with AD. These findings highlight the complementary nature of EC and EO conditions in assessing and differentiating AD cohorts.

**Conclusion:**

Our results underscore the potential of utilizing low-density EEG data from resting-state paradigms, combined with machine learning techniques, to improve the identification and classification of AD.

## Introduction

Alzheimer’s disease (AD) is a neurological disorder characterized by progressive neurodegeneration and synaptic dysfunction. The degeneration leads to decline in cognitive and behavioral functions, which ultimately interferes with the individual’s daily life. The early and accurate diagnosis of AD is of utmost important as it empowers AD patients and their families to understand the disease and explore available palliative therapies (Association A.s, 2009; Livingston et al., 2020). Furthermore, the future advent of disease-modifying treatments for dementia underscores the urgent need to identify reliable and easily accessible tools for diagnosing AD (Cummings et al., 2007; Cova et al., 2017; Cruzat et al., 2023; Prado et al., 2023).

The diagnosis of AD in clinical setting indeed relies primarily on cognitive, biochemical, and neuroimaging markers. These measurements are often obtained through techniques such as positron emission tomography (PET) and magnetic resonance imaging (MRI) in research setting (Dubois et al., 2007; Prado et al., 2022). However, it’s important to acknowledge that while the accuracy of AD diagnosis based on these markers ranges from 85 to 90%, it requires experienced clinicians, meticulous and exhaustive testing sessions, as well as costly and limited access to neuroimaging tools and invasive procedures (Sarazin et al., 2012). These constrains significantly hinder the widespread implementation of early AD diagnosis, particularly in low-income countries, remote and rural regions, and even in metropolitan areas with long wait times for non-emergency MRIs appointments, which can stretch to several months (Barua et al., 2014; Mukadam et al., 2024). Therefore, there is a critical need to develop alternative and more accessible diagnostic approaches, accompanied with the utilization of computerized algorithms, to enhance the early and accurate detection of AD.

Electroencephalography (EEG) is a non-invasive and cost-effective technique used to study of neurodegenerative disorders. Numerous studies employing EEG have identified characteristic features of AD, such as a shift in the power spectrum towards lower frequencies (Anaya et al., 2021), alterations in functional connectivity and phase synchronization between different brain regions (Briels et al., 2020), and reduced variability and complexity of brain activity (Takahashi, 2013). EEG has become an intriguing tool for studying and diagnosing AD. While current research often utilizes high-density electrodes setups in EEG studies, this approach may not always be feasible or optimal, particularly in clinical populations during research (Brito et al., 2019; van den Munckhof et al., 2018) and diagnostic testing (Aeby et al., 2021; Cassani et al., 2017). However, there is a growing trend towards large-scale neuroscience studies and the implementation of community-based approaches, with a focus on precision medicine using brain-based biomarkers. This emerging trend may pave the way for the integration of low-density electroencephalography (EEG) in future research endeavors. The availability of novel, portable, cost-effective, and user-friendly EEG systems within clinical settings holds promise as a valuable tool for assessing older individuals at risk of developing cognitive disorders, particularly in resource-limited regions. Previous investigations have demonstrated that these systems can reliably capture resting-state EEG activity with satisfactory signal-to-noise ratio across both young and elderly populations (McWilliams et al., 2021; Troller-Renfree et al., 2021; Rogers et al., 2016). Nevertheless, the significance of the observed differences between healthy control (HC) individuals and seniors with AD remains unclear at both the group and individual levels when using a limited number of electrodes to extract EEG biomarkers for statistical models. Additionally, EEG recordings in the eyes closed (EC) resting state are commonly used in AD studies, while the procedure of keeping the eyes open (EO) is not widely employed in dementia research, despite being routine in clinical neurophysiology. Interestingly, AD cohorts have shown significant impairment, such as reduced reactivity of posterior alpha rhythms (Babiloni et al., 2022; Babiloni et al., 2010; Babiloni et al., 2019), during EO, highlighting the need for further investigation into EO datasets and their potential contribution to understanding AD.

To this end, we utilized an 8-channel EEG acquisition system to capture resting-state EEG from individuals with AD and HC under both EC and EO conditions. We then conducted a comprehensive evaluation and comparison of various features, including power spectrum, phase-locked values, weighted lag phase index, and frequency-coupling values between the AD and HC groups. Our aim was to investigate whether any of these EEG features or combinations of features could server as clinical biomarkers using low-density EEG devices. We hypothesized that combining EEG features with eye-states would result in a more effective distinction between the AD and HC groups compared to utilized features from single eye state alone.

## Methods

### Participants

Sixty-two participants were recruited from the Department of Neurology, the First People’s Hospital of Qin Huang Dao. Participants were evaluated by a panel of cognitive neurologists and fulfilled clinical diagnostic criteria for AD (Mueller et al., 2005). Healthy controls were required to have a Mini-Mental State Examination (MMSE) score of 26 or above and a Clinical Dementia Rating (CDR) score of 0. We excluded individuals with a history of alcohol or drug abuse, current or known history of major depression or other neuropsychiatric conditions such as psychosis. Patients who were receiving medications known to affect brain activity, those with severe psychological distress and comorbid neurodegenerative diseases like Parkinson’s disease were also excluded. To minimize potential interference with EEG results, participants were instructed to avoid consuming caffeine and alcohol for 24–48 h prior to data collection. Additionally, participants were required to get adequate sleep and avoid staying up late before the session. The study was conducted in accordance with the Declaration of Helsinki and approved by the ethics committee of the First People’s Hospital of Qin Huang Dao. Written informed consent was obtained from each participant prior to recruitment into the study.

The neuropsychological battery was performed by trained psychologists who evaluated global cognition. Global cognition was assessed using the MMSE and Montreal Cognitive Assessment (MoCA) tool (Nasreddine et al., 2005). Out of 62 participants, we included 55 participants in the analysis (26 subjects with AD and 29 HC older adults) who passed quality control of EEG data (see EEG Acquisition and Preprocessing section). Both groups were well-matched in terms of demographics including age, gender, and handedness, education level.

### EEG data acquisition and preprocessing

EEG data were collected with an active EEG-system (JL - EEG8w), developed by the State Key Laboratory of Cognition and Learning of Beijing Normal University. Data were recorded at 1 kHz sampling rate with 0.1–80 Hz. According to the 10–20 international standard electrode system, 8 electrode position was used, i.e., F3, F4, T3, T4, C3, C4, O1 and O2. The impedance of each active electrode was controlled below 100 kΩ. Resting-state EEG were recorded during two 5-min blocks (one with EC and the other one with EO) in a random order. Participants sat in a comfortable chair, kept quiet and relaxed, and kept their bodies motionless or minimized as much as possible to reduce artifacts. During the eyes-open state, participants were instructed to focus steadily on a small cross displayed on the computer screen in front of them, a common method used to mitigate eye movements (Boytsova and Danko, 2010; Barry and De Blasio, 2017). They were instructed to sit still and minimize blinks or eye movements.

EEG data were preprocessed and analyzed offline using the MATLAB 2020b (The MathWorks, Natick, MA, USA), the Harvard Automated Processing Pipeline for EEG (HAPPE), and the EEGLAB toolbox. The EEG data were first filtered from 0.1 to 45 Hz, the EEG data was resampled to a frequency of 250 Hz. The original data were referenced to the Cz electrode and the filtered EEG data were then re-referenced to the average reference. Automatic artifact detection algorithms are applied to identify and remove segments of the data that contain unwanted artifacts such as eye movement and blinks, breathing, and muscle activity. Specially, standard HAPPE processing were employed to reject artifacts, designed for the preprocessing pipeline of low-density EEG configurations (Gabard-Durnam et al., 2018). The HAPPE artifact removal steps included bad channel identification, electrical line noise removal via Cleanline multitapering approach, artifact removal through wavelet-enhanced ICA and followed by a second ICA decomposition with automated component rejection above 50% artifact probability via the Multiple Artifact Rejection Algorithm (Winkler et al., 2014). Bad channels were then interpolated and EEG data were re-referenced to the average reference and mean signal detrended. Manual inspection of the remaining data is also important to ensure the accuracy of the analysis, participants with a bad channels or with <4 - s epochs were discarded. For each subject, the time series were divided into 75 sample (4 - s) segments. During the EO condition, the mean number of segments removed due to artifacts (mean ± std) was 13.12 ± 4.83 for the AD group, and 8.53 ± 3.61 for the HC group. During EEG data collection, the EC and EO conditions were randomized for each participant, allowing for the formation of an average for each condition. To eliminate potential possible average or order effects and ensure equalization of the number of epochs for each participant and condition, we retained the 2 min of EEG data (30 epochs) from the middle of each condition for further analysis. Seven participants were excluded for excessive artifacts (fewer than 30 epochs for at least one condition).

### EEG analysis

#### Power spectrum

As mentioned in the above preprocessing section, the EEG signal has been divided into 30 epochs of 4 s data segment. The 250 points (1.0 s) Hamming window was used to slide each data in the 100 points (0.4 s) step, the overlap rate is 0.6. Calculate the Fourier transform of 1,024 points to obtain the estimated power spectrum for each data point. Before calculating the Fourier transform of the EEG data, we applied data mirroring at the beginning and end of each 30-epoch segment, and the first and last epochs were replicated at the start and end of the segment to mitigate edge artifacts introduced by the Hamming window, respectively.

The whole brain power spectrum of all participants were calculated in both groups, perform group averaging (Cassani et al., 2017), and calculated the absolute power spectrum in the following four frequency intervals: delta (0.1–4 Hz), theta (4–8 Hz), alpha (8–13 Hz), beta (13–30 Hz) (Farina et al., 2020). In this study, we did not consider gamma oscillation, as the EEG in this frequency band is easily contaminated by muscle artifacts. Furthermore, the relative power of each frequency band was calculated by dividing the absolute power of each band by the sum of the absolute power of 1-45 Hz (Kwan et al., 2018).

#### Phase locked value (PLV)

Phase locked value is a metric used to quantify synchronous trends in EEG signals (Krusienski et al., 2012). The advantage of PLV is that it can measure the phase and amplitude components separately for a given frequency range. In the case of repeated stimulation, the latency period of phase synchronization or slight phase change in the PLV measurement test is observed. To calculate the phase synchronization of two EEG signals, the PLV calculation program calculates the instantaneous phase difference between signals within a specified frequency band. The synchronous measurement PLV at time t is defined as shown in Equation (1):

(1) $$PLV=\frac{1}{N}|\overset{N}{\underset{n=1}{\sum }}\exp\left\{j\left(\Delta \varphi \left(t,n\right)\right)\right\}|$$

Where N is the total number of tests, Δφ (t, n) = φ1 (t, n) - φ2 (t, n) is the instantaneous phase difference between signals.

In most EEG studies, PLV was used to measure the inter experimental variability of phase at time t. If there is no significant phase change during the test, the PLV approaches 1, otherwise it may be zero (Shan et al., 2022).

We sequentially calculated the phase-locking value (PLV) between the two electrodes by performing Hilbert transform on the respective electrode signals, extracted their imaginary parts, subtracted the phase, and subsequently computing PLV. Finally, we averaged PLV values from 30 epochs to obtain the final PLV characteristics.

#### Weighted phase lag index (wPLI)

The weighted phase lag index (wPLI) is an extension of the phase lag index (Šverko et al., 2022). PLI represents the asymmetry of the instantaneous phase distribution between two signals. The weighted version of PLI is defined as phase leading or lagging, weighted by the amplitude of the imaginary part of the complex cross spectrum. This limits the parasitic phase coupling around the origin caused by small disturbances (Tillem et al., 2018).

Each indicator of weighted phase lag is characterized by the distribution of phase angle difference (Xing et al., 2017). The instantaneous phase lag and amplitude can be obtained through cross power density spectra. Cross power density is defined as shown in Equation (2):

(2) $$S_{xy}\left(\omega \right)=\lim_{T\to \infty }\frac{1}{T}\exp\left\{Y_{x}^{*}\left(\omega \right)Y_{y}\left(\omega \right)\right\}$$

Where $S_{xy}$ is the cross spectral density function between the signals $Y_{y}\left(t\right)$ and $Y_{x}\left(t\right)$ . Signal $Y_{x}\left(\omega \right)$ is signal $Y_{x}\left(t\right)$ in *ω* Finite Fourier Transform at Frequency, $Y_{x}^{*}\left(\omega \right)$ is Complex conjugate of $Y_{x}\left(\omega \right)$ . Cross power density should be applied to each frequency band of interest (0.1 Hz–30 Hz).

The distribution of phase angle difference can face the positive or negative side of the composite plane (Cohen, 2015; Vinck et al., 2011). The more concentrated the phase angle difference is on the same side, whether it is positive or negative, the higher the phase lag synchronization will be (Cohen, 2015). WPLI is defined as shown in Equation (3):

(3) $$wPLI_{xy}=\frac{\frac{1}{n}\sum_{t=1}^{n}|\mathrm{imag}\left(S_{xyt}\right)|sgn\left(\mathrm{imag}\left(S_{xyt}\right)\right)}{\frac{1}{n}\sum_{t=1}^{n}|\mathrm{imag}\left(S_{xyt}\right)|}$$

Where $\mathrm{imag}\left(S_{xyt}\right)$ represents the cross spectral density at time point t in the complex plane xy , and sgn represents a symbolic function (−1, +1 or 0).

We sequentially computed the wPLI between two electrodes by segmenting the electrode signal involved in the calculation into 10-s windows with a 50% overlap. To achieve this, we employed the multi - frequency transformation method for multi-carrier frequency decomposition of the data at intervals of 0.1 Hz, followed by wPLI computation between the respective channel pairs.

#### Frequency coupling

To compare the changes near the alpha frequency band of the test subjects, we derived a novel feature by calculating the ratio of relative power between the theta and beta frequency bands. This ratio operation on both frequency bands yields the coupling value (theta/beta) represent their interplay (Miao et al., 2021). The magnitude of this value indicates the relative energy distribution between the theta and beta frequency bands, with values greater than 1 suggest a higher energy concentration in the theta band, and vice versa for the beta band. Consequently, we employed this feature to quantify near the alpha frequency band alterations in our subjects.

### Support vector machine (SVM)

Support vector machine (SVM) is a popular machine learning algorithm that is used for classification and regression analysis (Durongbhan et al., 2019). The algorithm works by finding the optimal boundary or hyperplane that can separate the data into different classes. The SVM algorithm can handle both linear and nonlinear classification problems (Fröhlich et al., 2021). In the case of nonlinear classification, the SVM algorithm uses a kernel function to map the data into a higher dimensional space, where the optimal boundary can be found (Cao et al., 2022). The commonly used kernel functions are linear, polynomial, and radial basis kernels. In this study, the linear kernel was found to be the optimal kernel function for the SVM model, based on its performance compared to the other kernels tested. In order to avoid possible overfitting of the model and obtain a reliable performance of the proposed model. We applied k - fold cross validation (CV) technique to all the classifiers. The entire dataset testing was randomly divided into k folds of equal size. For each fold, the k − 1 subsets were applied for training and the remaining one subset was applied for testing. This process was repeated for k − 1 more times. The overall performance of each classifier was evaluated by calculating the average result of k folds. In this study, we selected *k* = 10. In order to improve the performance of the classifier, a 10-fold cross-validated linear kernel SVM was selected in this study, and features were screened in two rounds, namely Mann–Whitney U test and single-feature classification accuracy of 70%.

### Statistical analysis

Two-sample t test was used to compare age differences, Chi - square test was used to compare sex differences, education levels and conventional hands between AD group and HC group. To examine the differences between groups, we used a linear regression model with EEG features as effects of interest, while age, sex, and education level were used as covariates. Residuals were analyzed using the Mann–Whitney U test, and EEG features were subsequently corrected for multiple comparisons using the Bonferroni method. The difference in relative power of oscillation, PLV, wPLI, and frequency coupling value, *p* < 0.05, is statistically significant.

## Results

### Group differences in demographic and clinical characteristics

There were no significant group differences in age, gender, education levels and handedness. However, clinical test scores differed between AD and HC groups (Table 1). AD patients had significantly lower MMSE and MoCA scores than the HC group (MMSE: *t* = −7.372, *p* < 0.001; MoCA: *t* = −11.712, *p* < 0.001).

**Table 1:** Subject demographic and clinical characteristics.

	AD (*N* = 26)	HC (*N* = 29)	*p*-value
Age(years)	69.35 (10.08)	64.17 (10.64)	—
Gender(M/F)	11/15	12/17	—
Handedness(L/R)	0/26	0/29	—
Education(P/J/S/A/U)	4/2/13/2/5	5/2/15/3/4	—
MMSE	15.45 (9.03)	29.39 (0.89)	<0.001
MoCA	12.05 (6.48)	28.22 (1.98)	<0.001

Values represent mean (standard deviations). Group difference in age, MMSE, and MoCA were evaluated with independent-sample t test, while those of gender, handedness and education were evaluated with Chi-square test. N, number of subjects; HC, healthy controls; AD, Alzheimer’s disease; P, Primary school; J, Junior high school; S, Senior middle school; A, Associate College; U, University; MMSE, Mini-Mental State Examination; MoCA, Montreal Cognitive Assessment.

### Group differences in EEG characteristics

#### Whole-brain power spectrum

**Figure 1:**
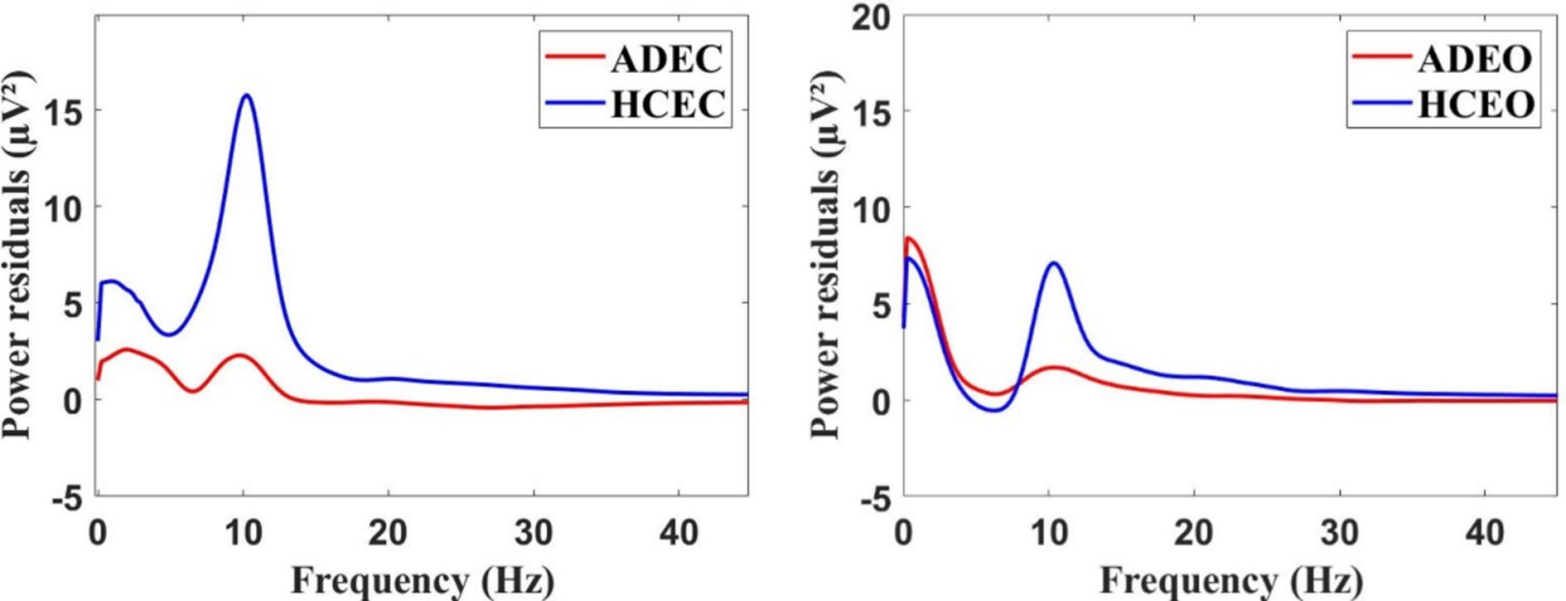
The grand average of the EEG power spectrum residuals for AD and HC groups during the resting state, both in the EC (eye closed) condition (left) and EO (eye open) condition (right).

Figure 1 showed the distribution of EEG power across different frequency bands for both AD and HC during EC and EO. The power spectrum during EC showed a significant difference (Mann–Whitney U test, *p* < 0.001) between the two groups in the alpha frequency band, with AD patients showing a lower peak frequency compared to HC. Specifically, the peak frequency of AD patients was 9.76 ± 0.34 Hz, while that of HC was 10.09 ± 0.65 Hz (Mann–Whitney U test, *p* < 0.05). However, no significant difference was observed in the corresponding frequency band between the two groups during EO condition.

#### Functional connectivity

**Figure 2:**
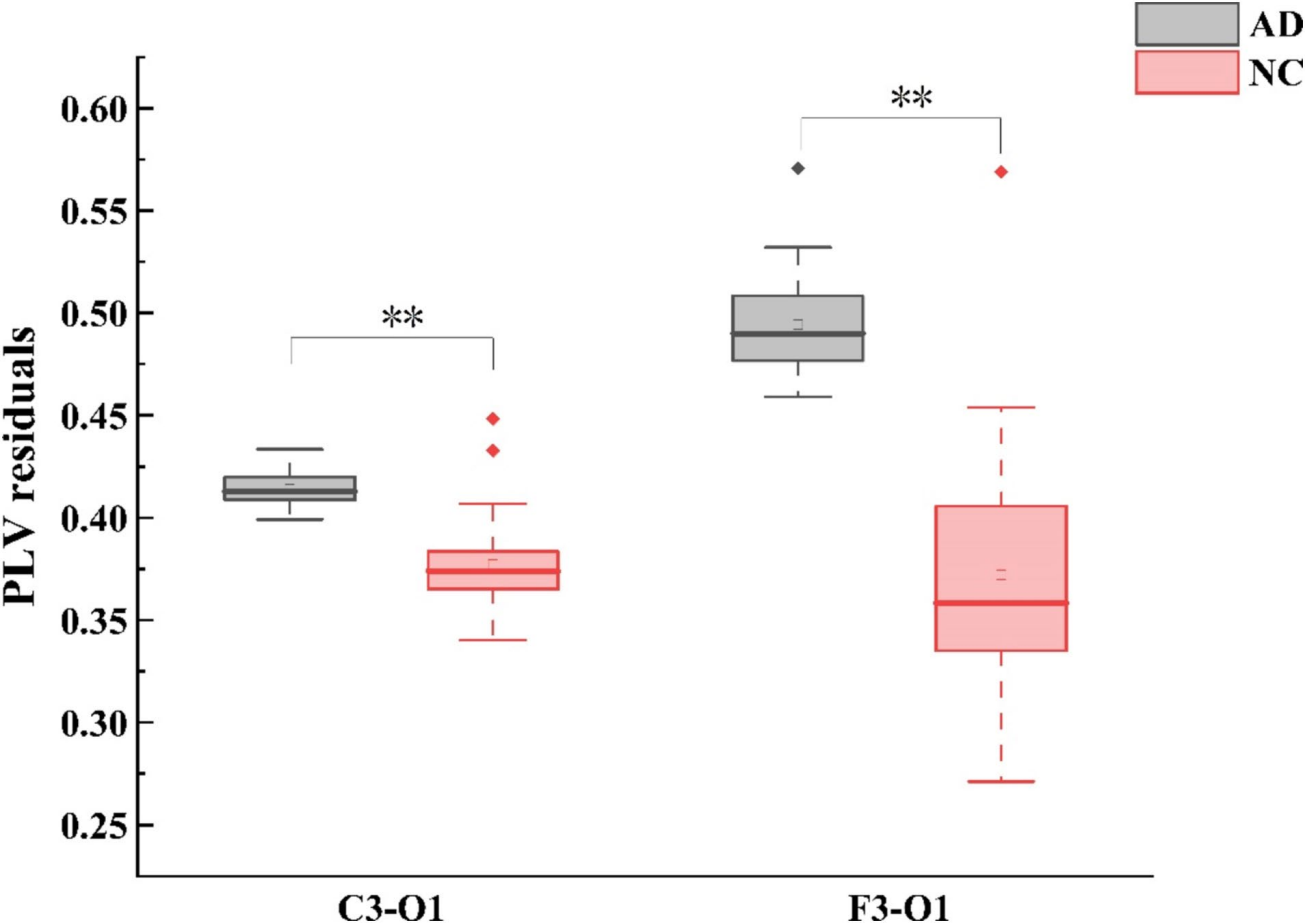
Alterations in theta-band PLV (phase locking value) connectivity residuals of the occipital region in individuals with AD compared to HC subjects. Statistically significant group difference was indicated by * (*p* < 0.05, with Bonferroni correction for multiple comparison).

In terms of PLV, group differences were observed in the theta frequency band during EC. Compared with HC, AD patients exhibited higher left fronto-occipital (F3 - O1: Mann-Whitney *U* test, *p* < 0.001) and centro-occipital (C3 - O1: Mann-Whitney *U* test, *p* < 0.001) PLV as shown in (Figure 2). Correspondingly, no group difference was found during EO for each frequency band. The inter-electrode differences of PLV between EC and EO conditions were further analyzed, AD patients had lower values in the left fronto-right temporal region (F3 - T4: Mann–Whitney *U* test, *p* < 0.001).

**Figure 3:**
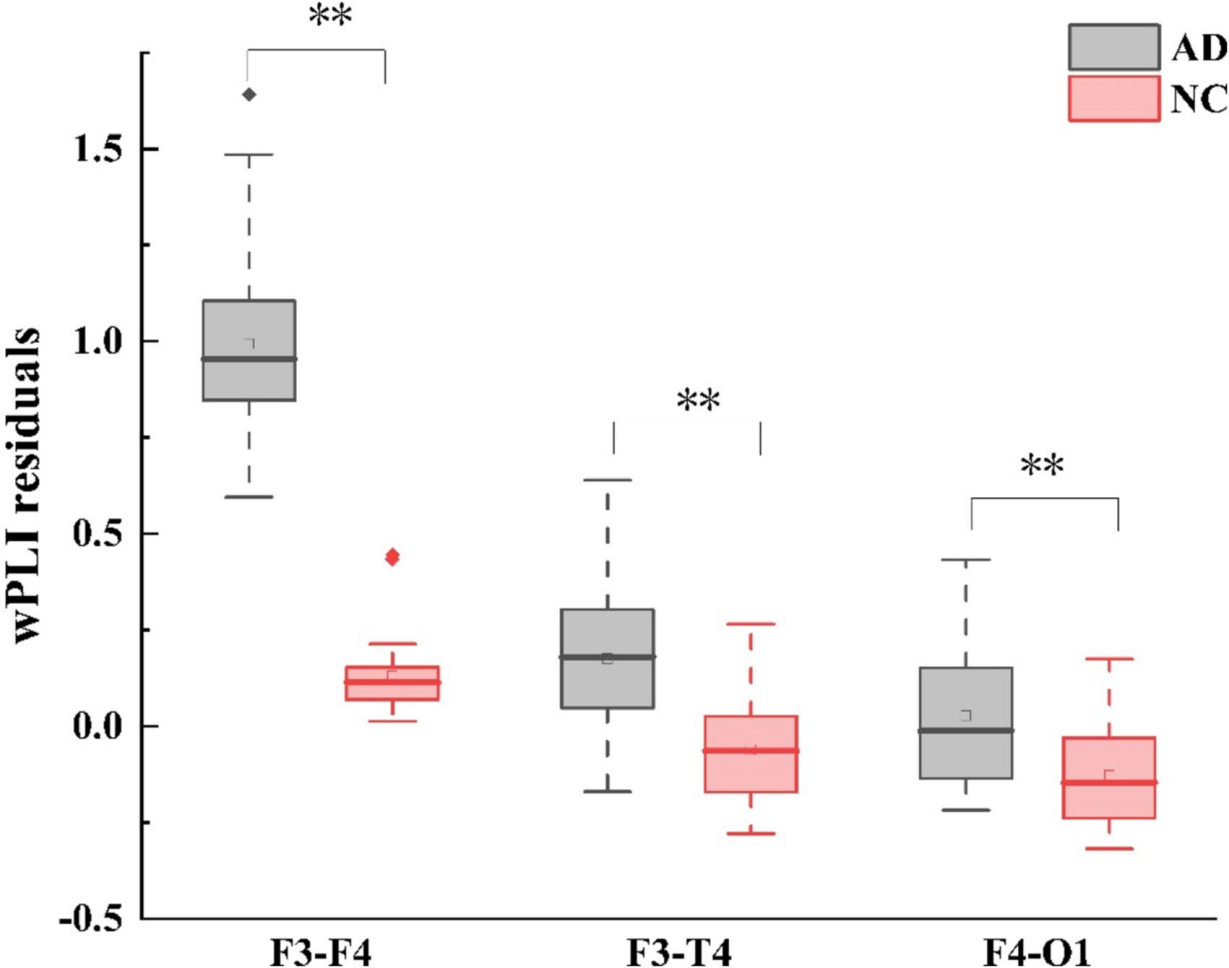
Alterations in theta-band wPLI (weighted phase lag index) connectivity residuals of the frontal region in individuals with AD compared to HC subjects. Statistically significant group difference was indicated by * (p < 0.05, with Bonferroni correction for multiple comparison).

In terms of wPLI, group differences were also observed in the theta frequency band during EC. Compared with HC, AD patients exhibited higher left fronto-right frontal (F3 - F4: Mann-Whitney *U* test, *p* < 0.001), left fronto-right temporal (F3 - T4: Mann-Whitney U test, p < 0.001), and right fronto-left occipital (F4 - O1: Mann-Whitney *U* test, *p* < 0.001) wPLI as shown in (Figure 3). Correspondingly, no group difference was found during EO for each frequency band. For the differences of wPLI under EC - EO conditions, AD patients had significant difference in the left fronto-right temporal (F3 - T4: Mann–Whitney *U* test, *p* < 0.001) and right center-temporal (C4-T4: Mann–Whitney *U* test, *p* < 0.001) regions (Figures 2, 3).

#### Frequency coupling

**Figure 4:**
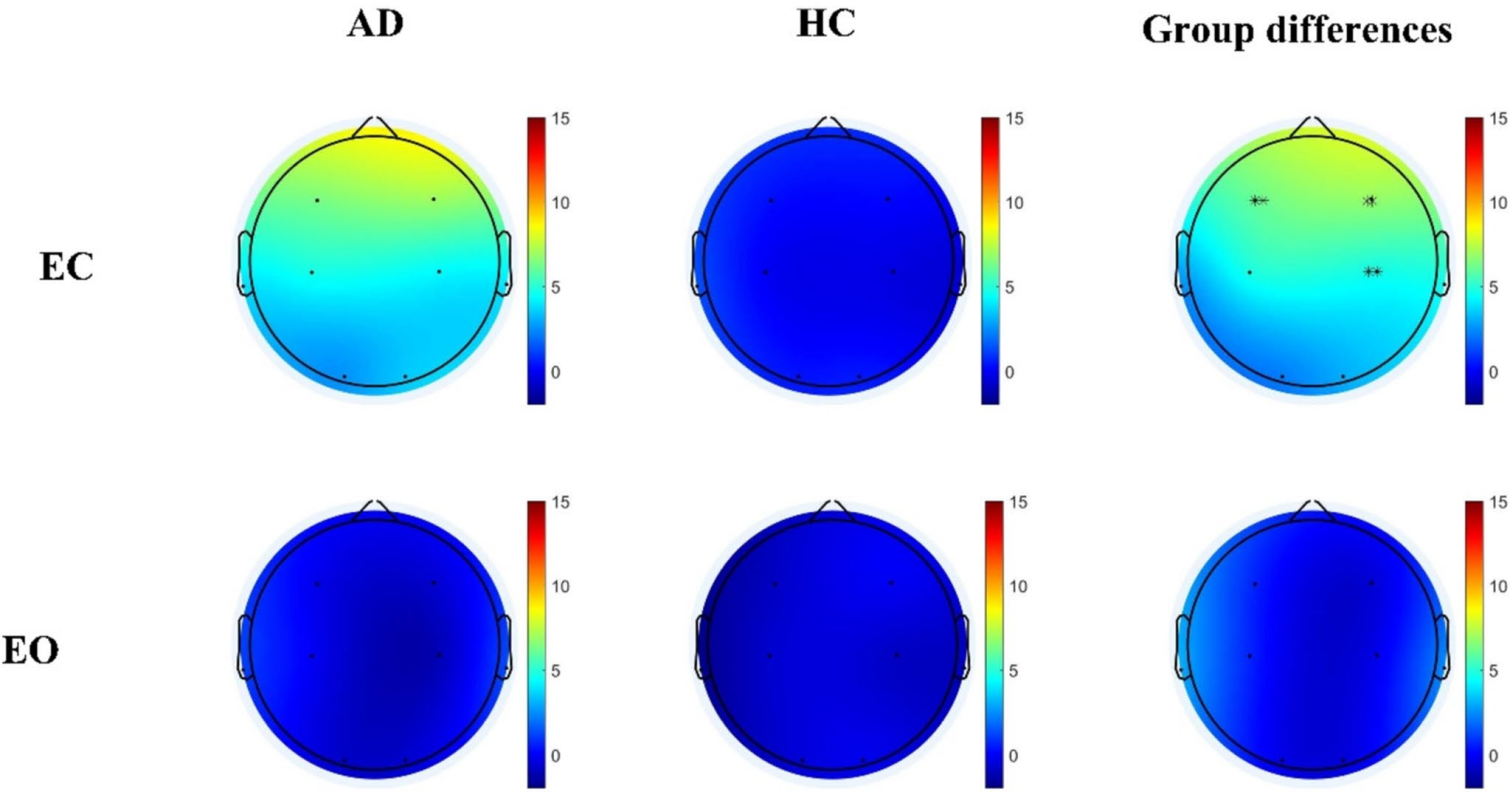
Differential theta/beta frequency coupling residuals in individuals with AD relative to HC during both EC and EO conditions. Statistically significant group difference was indicated by * (*p* < 0.05, with Bonferroni correction for multiple comparison).

The spatial distributions of the average frequency coupling (theta / beta) for AD patients and HC during EC and EO were shown in Figure 4. During EC, AD patients exhibited significantly higher coupling values in the frontal and central lobes compared to HC (F3: Mann–Whitney *U* test, *p* < 0.001, F4: Mann–Whitney *U* test, *p* < 0.001; C4: Mann–Whitney *U* test, *p* < 0.001). However, no significant group differences were observed during EO. In order to further reduce the impact of volume conduction, we proposed to use frequency coupling to conduct a comparative analysis of AD and HC, EC - EO condition characteristics (F4: Mann–Whitney *U* test, *p* < 0.001).

### Classification comparison of two paradigms

After two filtering steps, the retained features were combined into a feature group. The Combined Conditional Characteristics (EC-EO) were derived by subtracting the EEG signature of the corresponding EO from that of the EC. Feature selection for the combination underwent two rounds of screening, namely Mann–Whitney U test, followed by a classification accuracy threshold of 70% for each individual feature. The final selected features included PLV for left fronto-right temporal region, wPLI for both left fronto-right temporal and right center-temporal regions, and frequency coupling (theta/beta) for the right frontal lobe. The obtained feature group was fed into a linear kernel support vector machine (SVM) and classified using 10-fold cross validation. The classification indicators obtained by the two groups of participants were compared and listed in the table below.

The classification performance based on single condition (EC) and combined conditions (EC - EO) were evaluated, with the letter showing improved performance. Under the combined conditions, the accuracy, sensitivity, and specificity were found to be 96.36, 98.10, and 97.78%, respectively. These values showed an improvement of 3.63, 5.77 and 4.45% respectively, when compared to the single normal form. In this study, a random forest (RF) classifier was also used, and the accuracy, sensitivity, specificity, and AUC of RF under the combined conditions were 94, 92.00, 96.00, and 95.5%, respectively. These results indicate that the SVM method has higher accuracy, sensitivity, specificity, and AUC than RF. Detailed RF results are provided in the Supplementary material.

### ROC curves of two paradigms

**Figure 5:**
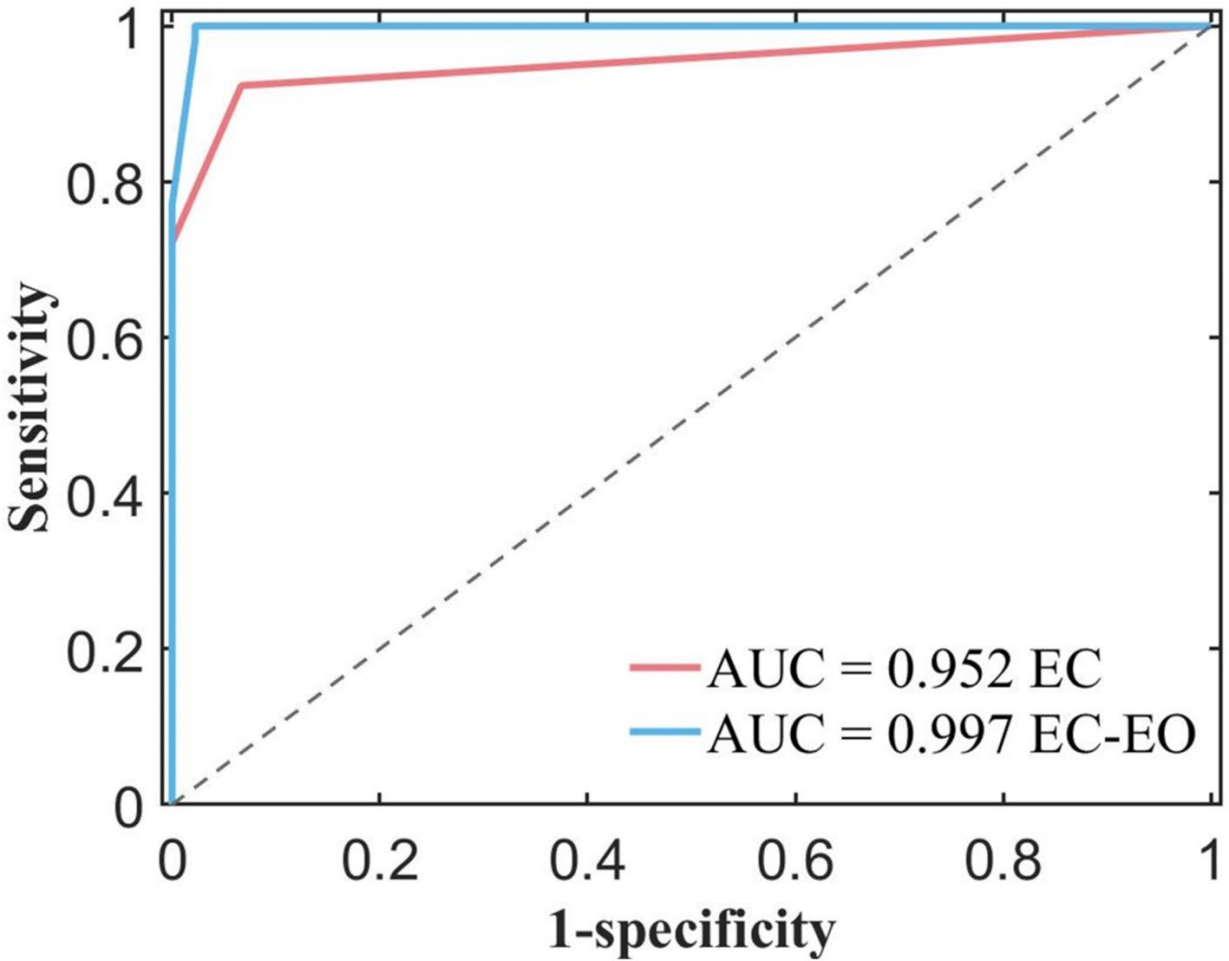
ROC curves and AUC values for different feature constructions extracted from the EC (eye closed) condition only and EC combined with the EO (eye open) condition.

In order to compare the performance of classifiers obtained using feature groups under two different paradigms, we plotted the ROC curves of the two classifiers in Figure 5. From the graph, it was observed that the blue ROC curve (EC - EO) was located above the red curve (EC), and the AUC of the blue curve was closer to 1, indicated that the classifier trained using normal form combined with features had better classification performance.

As shown in Table 2, AUC for the single condition was 0.9520, and AUC for the combined conditions was 0.9970. Compared to the single EC conditions, the classifier obtained by combining EC and EO conditions with the feature group had better classification performance. It indicated that the features combined with EC and EO conditions had better classification potential compared to the single form, and were expected to become powerful biomarkers. These findings suggest that these combined features have the potential to serve as powerful biomarkers.

**Table 2:** Classification performance comparison between different feature construction schemes for distinguishing AD from HC.

	EC	EC + EO
ACC	92.73%	96.36%
Sensitivity	92.33%	98.10%
Specificity	93.33%	97.78%
AUC	0.952	0.997

## Discussion

The present study employed low-density EEG to identify potential biomarkers that can effectively differentiate between two distinct populations. During the EC condition, decreased alpha power in the AD group. Both analysis of PLV and wPLI in the theta band indicated that the alterations in the AD brain network predominantly involved in the frontal region. In addition, the AD group had increased frequency coupling in the frontal and central regions. Furthermore, decreased theta band functional connectivity within the fronto-central lobe and increased frequency coupling in frontal region were found in AD group from EC to EO. More importantly, the combination of EC and EO quantitative EEG features improved the inter-group classification accuracy when using support vector machine (SVM) in older adults with AD. Our findings highlight the promise use of low-density EEG data from resting-state paradigms combined with machine learning techniques in enhancing our understanding and diagnosis of AD.

Both the AD and HC groups exhibited a noticeable peak in the alpha band during the EC condition compared to EO. This distinct peak can be attributed to activation of the visual system, which is more prominent during EC (Barry et al., 2007). Consistently, AD patients showed a decrease in average power spectrum, primarily within in alpha frequency band during EC (Blackburn et al., 2018). In patients with AD, the power of low-frequency alpha waves is significantly reduced compared with normal older adults, reflecting the gradual weakening of the function of the thalacortex and cortical systems in controlling visual attention (Arnáiz and Almkvist, 2003). This finding echoes the clinical manifestations of visuospatial deficits in AD patients (Arnáiz and Almkvist, 2003). Further studies have suggested that this significant reduction in low-frequency alpha wave power may be due to damage to the cholinergic pathway, which affects cerebral blood flow and thus affects the improvement of attention and memory function (Claassen and Jansen, 2006). In addition, the AD group demonstrated a lower peak frequency in the alpha band compared to the NC group, supporting the pathological shift of oscillatory power from higher to lower frequencies in AD patients (Meghdadi et al., 2021).

Cross-frequency coupling plays a crucial role in coordinating perception, memory, consciousness, and other cognitive processes (Palva and Palva, 2007; Wang et al., 2014). Theoretical perspectives propose four models for cross-frequency coupling: phase-to-amplitude, power-to-power, phase-to-phase, and phase-to-frequency interactions (Abubaker et al., 2021). While some studies have explored changes in phase-to-power coupling in AD (Goodman et al., 2018; Musaeus et al., 2020), our study focuses on alterations in power-to-power coupling in AD. We discovered that the increase in theta activity can be accompanied by a decrease in beta activity, resulting in an increase in theta/beta coupling during the eye closed (EC) condition. This finding aligns with previous studies on phase-to-power coupling in AD (Wang et al., 2017), indicating a disruption of coupling between frequency bands in AD patients. Moreover, the spatial distribution of theta/beta coupling revealed predominant alterations in the frontal and central regions, suggesting a potential underlying mechanism for the deficits in these brain areas. Notably, this is the first study to analyze alterations in power-to-power coupling in AD. These findings, along with our power spectrum results, provide further evidence of the pathological shift of oscillatory power from higher to lower frequencies in AD.

Both the PLV and wPLI calculated in theta band revealed functional network (FC) differences between the AD and HC groups, particularly in the frontal lobe. Although PLV and wPLI belong to the same category of methods for evaluating FC strength based on phase lag, our study observed diverse changes in FC when using these two measures, which aligns with findings from other research (Chen et al., 2024; Močilnik et al., 2024). On one hand, wPLI exhibited higher sensitivity (Šverko et al., 2022), along with a higher standard deviation across individuals. On the other hand, in the AD group, PLV revealed an increase in inter-regional FC between frontal and occipital regions, while wPLI showed an increase in intra-regional FC within frontal lobe and an increase in inter-regional FC between occipital, temporal, and frontal regions. This discrepancy is related to the different calculation methods employed by these two measures. PLV quantifies the consistency of the phase difference between two signals. When the phase difference remains relatively constant over time, the PLV value is high, indicating strong phase locking or synchronization. Conversely, wPLI is designed to measure the degree of phase delay between two EEG signals, assigning weights to each phase difference based on the amplitude of the signals, which reduces the influence of phase differences that are close to zero (Vinck et al., 2011). Although PLV is more susceptible to volume conduction effects, the combined use of both measures in EEG classification study has demonstrated a significant improvement in classification accuracy (Duan et al., 2021), indicating that the integration of different methods can provide a more comprehensive understanding of brain connectivity. Given the potential limitations associated with volume conduction, our study implemented strategies to minimize its impact. We utilized a low-density EEG configuration with a minimum inter-electrode distance of 11 centimeters. This design helps mitigate volume conduction effects, which tend to be more pronounced when inter-electrode distances are less than 10 centimeters (Srinivasan et al., 2007). Additionally, our analysis primarily focuses on group-level differences rather than direct comparison of connectivity strength between electrode pairs within individual networks. Consequently, despite these differences, it is important to recognize that abnormalities in FC were expected to locate in the frontal lobe in the theta band. However, the PLV-based calculation method must still consider the issue of volume conduction, particularly for future studies involving low spatial resolution but high-density EEG. These findings support the presence of alterations in the frontal lobe and changes in low-frequency signals in individuals with AD (Fallon et al., 2017). In our study, AD patients exhibited a significant increase in wPLI in the theta band compared to HC, which is consistent with previous studies. Furthermore, a significant negative correlation between functional connectivity in the theta band and cognitive scores was found in AD patients (Yan et al., 2021), suggesting that increased theta band connectivity may be associated with more severe cognitive impairment in this population. These finding demonstrate that altered brain network connectivity, particularly in the theta band, may play a role in the cognitive decline observed in Alzheimer’s disease.

Prior to conducting the two sets of classification, a feature screening process was applied to the extracted frequency domain features and frequency coupling. It was found that incorporating frequency coupling in the EC condition, along with the previously mentioned features in both the EC and EO conditions, significantly enhanced classification accuracy compared to using only the PLV and wPLI in the EC condition as classification features. The substantial differences observed between the EC and EO conditions highlight the importance of incorporating experimental paradigms and their associated features as classification variables. This approach not only improves the accuracy and performance of the classification, but also allows for a more effective differentiation between the two groups. By combining multiple paradigms, the early diagnosis of AD can be facilitated (Kang et al., 2020).

The current study highlights the potential of utilizing low-density EEG data from resting-state paradigms, combined with machine learning techniques, to improve the identification and classification of AD, providing a promising approach for early diagnosis. Given the small sample size of our study, we chose SVM for classification due to their advantages in such scenario. SVM, through its regularization properties, effectively prevents overfitting and can extract meaningful features even with limited data, maintaining strong generalization capabilities (Cervantes et al., 2020). In contrast, random forest, as an ensemble learning method, improves prediction accuracy by combining the outputs of multiple decision trees. It is relatively easy to adjust and is suitable for handling large data sets. However, for small sample sizes, random forests can be affected by overfitting, especially if the number and depth of trees are not optimally adjusted (Breiman, 2001), on the other hand, neural networks are adept at capturing complex nonlinear relationships and are particularly well-suited for modeling complex data patterns. However, neural networks often require large amounts of training data to perform well, and on small datasets, neural networks are highly sensitive to network architectures such as the number of layers and neurons, which makes training on small datasets easy to overfit (Schmidhuber, 2015). Therefore, considering our research objectives and the current sample size, we believe that SVM is the most appropriate choice. Compared with other models, SVM significantly improves the robustness of our classification method, effectively avoids overfitting, and provides more reliable classification results.

However, the small sample size in this study increases risk that the model may capture noise and specific patterns from the training data rather than generalizable features, which can lead to diminished performance on unseen data. Additionally, a limited number of samples may result in unstable parameter estimates, adversely affecting the model’s predictive accuracy. In terms of generalization capability, the small sample size may not adequately represent the diversity of the target population, thereby limiting the model’s applicability to broader clinical contexts. While we believe that our choice of SVM mitigates some risks associated with small sample sizes through its regularization properties (Cervantes et al., 2020), we agree that future studies should aim to utilize larger datasets to validate our findings and enhance the robustness of the results.

## Limitations and future work

This study has a few limitations that should be addressed in future research. Firstly, the analysis did not include individuals with mild cognitive impairment (MCI), which is an intermediate stage between normal aging and AD. While the current study primarily aims to confirm the electrophysiological markers for identifying AD, the absence of an MCI group limits our ability to explore the transition from normal cognition to the early stages of AD. Future research is planned to include an MCI group to study the continuity of cognitive decline leading to AD and to enhance early diagnostic efforts. Secondly, we could not conduct comprehensive neuropsychological tests on the participants. While our study focused on the diagnostic potential of EEG features, this limitation prevented us from establishing correlations between the EEG features and the cognitive performance, which could have potentially improved classification accuracy. Future studies could explore whether different EEG changes associated with specific cognitive functions exhibit varying classification efficacy, or investigate the potential of combining EEG data with specific cognitive classifications. Thirdly, the small sample size of 26 AD patients and 29 healthy controls may restrict the generalizability of our findings. Future studies should utilize larger datasets to validate these results and enhance the robustness of the conclusions drawn. Additionally, incorporating advanced feature extraction methods, such as nonlinear dynamic features and network analysis, will help to further elucidate changes in EEG activity in AD patients. In summary, future studies should aim to address these limitations by including MCI individuals and conducting additional neuropsychological tests prior to EEG data collection. By implementing these measures, more biomarkers can be identified that effectively distinguish AD from healthy controls HC, ultimately leading to more accurate early diagnosis of AD.

## Conclusion

In conclusion, our study identified the pathological characteristics of patients with AD using low-density EEG. The integration of multiple experimental paradigms led to improved classifier performance and enhanced classification accuracy compared to using a single paradigm alone. Future research should focus on incorporating new experimental paradigms, including EEG signals from individuals with MCI, and comprehensively investigate the progression from HC to AD for effective early diagnosis.

## Data Availability

The original contributions presented in this study are included in this article/Supplementary material, further inquiries can be directed to the corresponding author.
