# Supplementary material for: Alzheimer’s disease diagnosis using rhythmic power changes and phase differences: a low-density EEG study
Source: Front Aging Neurosci. 2025 Jan 17;16:1485132. doi: 10.3389/fnagi.2024.1485132 (PMC11782140; doi:10.3389/fnagi.2024.1485132)
Supplement: Supplementary file 1 [file Data_Sheet_1.docx]

**Supplementary Information**

**Methods and Results of the Random Forest Classifier**

Random forest (RF) developed by Leo Breiman in 2001 has been proved to be a powerful approach with excellent performance in classification tasks [1, 2]. The random forest uses bootstrap sampling technology to randomly select multiple subsamples from the original dataset and independently train a decision tree on each subsample. During the growth of the tree, a subset of features is randomly selected for node splitting, which effectively reduces the risk of overfitting the model. Eventually, the RF generates the final prediction by voting on the predictions of all trees (classification task) or averaging (regression task). This approach not only enhances the model's stability and accuracy, but also demonstrates effective handling of high-dimensional data and the assessment of feature importance [3]. In this study, grid search and 10-fold cross-validation were used for parameter tuning. The number of trees (N _trees_) that need to grow in each forest is 50, and the maximum depth of trees is 5. The number of attempts to select variables for splitting on each node (M _try_) that is considered at each split is a practical parameter because its optimal value depends on the data. Number of input variables (M _try_ ) use the default value (the square root of the number of input variables) [4].

The accuracy, sensitivity, specificity, and AUC of RF under the combined conditions were 94%, 92.00%, 96.00%, and 95.5%, respectively, while the accuracy, sensitivity, specificity, and AUC of the SVM method were 96.36%, 98.10%, 97.78%, and 99.70%, respectively. These results indicate that the SVM method has higher accuracy, sensitivity, specificity, and AUC than random forest.

**Table S1. Classification performance comparison between different feature construction schemes for distinguishing AD from HC.**

|  | **EC** | **EC+EO** |
| --- | --- | --- |
| **ACC** | **90.00%** | **94.00%** |
| **Sensitivity** | **92.00%** | **92.00%** |
| **Specificity** | **99.00%** | **96.00%** |
| **AUC** | **0.949** | **0.955** |


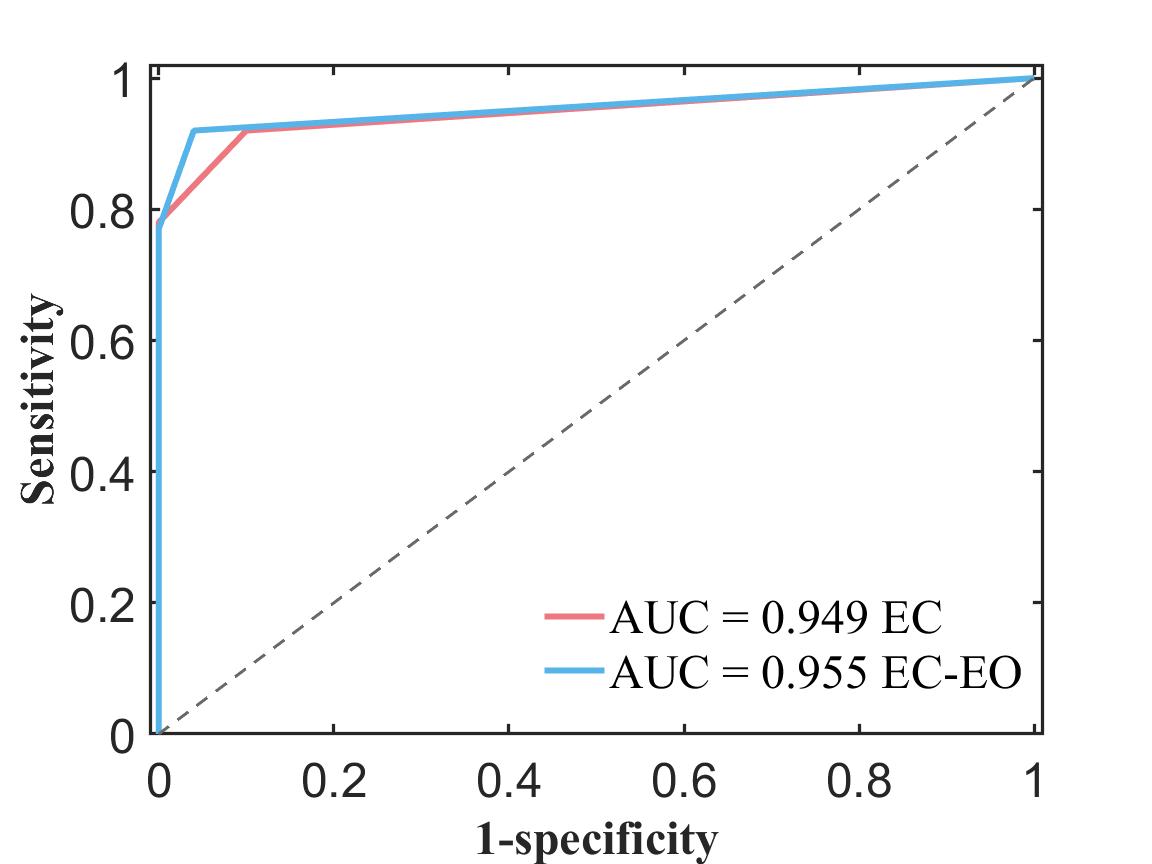


**Figure S1. ROC curves and AUC values for different feature residuals constructions extracted from the EC (eye closed) condition only and EC combined with the EO (eye open) condition by RF classification.**

References

1. Breiman, L., *Random Forests.* Machine Learning, 2001. **45**(1): p. 5-32.

2. Segal, M., *Machine Learning Benchmarks and Random Forest Regression.* Technical Report, Center for Bioinformatics & Molecular Biostatistics, University of California, San Francisco, 2003.

3. Chen, W., et al., *A random forest model based classification scheme for neonatal amplitude-integrated EEG.* BioMedical Engineering OnLine, 2014. **13**(2): p. S4.

4. Liaw, A., *Classification and regression by randomForest.* R news, 2002.
